# Personal Audiovisual Aptitude Influences the Interaction Between Landscape and Soundscape Appraisal

**DOI:** 10.3389/fpsyg.2018.00780

**Published:** 2018-05-22

**Authors:** Kang Sun, Gemma M. Echevarria Sanchez, Bert De Coensel, Timothy Van Renterghem, Durk Talsma, Dick Botteldooren

**Affiliations:** ^1^Department of Information Technology, Ghent University, Ghent, Belgium; ^2^Department of Experimental Psychology, Ghent University, Ghent, Belgium

**Keywords:** audiovisual interactions, landscape, soundscape, environmental perception, personal factor

## Abstract

It has been established that there is an interaction between audition and vision in the appraisal of our living environment, and that this appraisal is influenced by personal factors. Here, we test the hypothesis that audiovisual aptitude influences appraisal of our sonic and visual environment. To measure audiovisual aptitude, an auditory deviant detection experiment was conducted in an ecologically valid and complex context. This experiment allows us to distinguish between accurate and less accurate listeners. Additionally, it allows to distinguish between participants that are easily visually distracted and those who are not. To do so, two previously conducted laboratory experiments were re-analyzed. The first experiment focuses on self-reported noise annoyance in a living room context, whereas the second experiment focuses on the perceived pleasantness of using outdoor public spaces. In the first experiment, the influence of visibility of vegetation on self-reported noise annoyance was modified by audiovisual aptitude. In the second one, it was found that the overall appraisal of walking across a bridge is influenced by audiovisual aptitude, in particular when a visually intrusive noise barrier is used to reduce highway traffic noise levels. We conclude that audiovisual aptitude may affect the appraisal of the living environment.

## Introduction

The phrase ‘soundscape’ used in this study is as defined by International Organization for Standardization (ISO): an “acoustic environment as perceived or experienced and/or understood by a person or people, in context” (ISO, 2014). The subjective appraisal of our living environment is influenced by landscape and soundscape alike. It is well known that these influences are not independent. This interaction partly originates at a low level of auditory and visual perception. In soundscape theory, the importance of visual context on soundscape appraisal has been stressed (Weinzimmer et al., 2014; Botteldooren et al., 2015). Using virtual reality, it was likewise shown that the sonic environment affects overall pleasantness of the public space even when the participants in the experiment focused on visual designs and were kept unaware of the sound (Echevarria Sanchez et al., 2017). In the home environment, it has been shown that vegetation as seen through a window affects the self-reported noise annoyance at home (Li et al., 2010; Van Renterghem and Botteldooren, 2016; Leung et al., 2017). The visibility of a sound source may also affect the awareness of sound. On the one hand, it has been shown that people get more annoyed when the sound source is visible (Zhang et al., 2003), while other studies found that sound is actually less annoying when the source is visible (Maffei et al., 2013). It remains currently unknown what drives these differences. In this paper, we forward the hypothesis that a personal factor or multiple personal factors influence the interaction between landscape and soundscape appraisal. Personal traits and beliefs are known to influence the perception and appraisal of the sonic environment both at home [e.g., noise sensitivity (Miedema and Vos, 2003; Heinonen-Guzejev, 2009)] and in public spaces [e.g., meaning given to tranquility (Filipan et al., 2017) and recreation (Pilcher et al., 2009; Miller et al., 2014)]. So it is not unlikely that this additional personal factor would indeed exist.

Previous studies have already shown that considerable individual differences exist in the way humans process audiovisual information, ranging from differences in connectivity between auditory and visual pathways (e.g., van den Brink et al., 2013), to selective preferences in processing auditory or visual material (Giard and Peronnet, 1999). More generally, when engaged in a visual task, participants tend to ignore auditory stimuli, as demonstrated by the well-known Colavita effect (Colavita, 1974). One striking result from many studies on the Colavita effect is that when participants are presented with either auditory or audiovisual stimuli, and have to respond to a change in the auditory stimulus, they usually do so accurately on the auditory-only trials, but fail to detect this change when an audio–visual stimulus is presented to them. A main question is why participants miss such an auditory change.

One possible answer comes from Simons and Chabris, who explored how an unexpected object could go unnoticed during a monitoring task, in a phenomenon they described as inattentional blindness (Simons and Chabris, 1999). Recent research also demonstrates that a single discrete visual distractor can improve the detectability of an unexpected object in an inattentional blindness task (Pammer et al., 2014). Visual distractor processing tends to be more pronounced when the perceptual load of a task is low compared to when it is high [perpetual load theory (Lavie, 1995)]. Sandhu and Dyson studied the effect of auditory load on visual distractors and vice versa. They found that in both attend auditory and attend visual conditions, the distractor processing was evident, especially when the distractors were visual (Sandhu and Dyson, 2016). Perpetual load theory has been supported from assessing the impact of perceptual load on the flanker task (Eriksen and Eriksen, 1974), as well as behavioral paradigms, such as negative priming (Lavie and Fox, 2000), implicit learning (Jiang and Chun, 2001) and inattentional blindness (Cartwright-Finch and Lavie, 2007).

A possible explanation for inattentional blindness based on perpetual load theory is that conscious perception of task-irrelevant stimuli critically depends upon the level of task-relevant perceptual load rather than intentions or expectations (Cartwright-Finch and Lavie, 2007). Aging could increase the susceptibility to inattentional blindness (Graham and Burke, 2011). Likewise, individual differences in cognitive ability related to working memory and executive functions affect inattentional blindness (Fougnie and Marois, 2007). Several studies have shown that this phenomenon could be associated with general fluid intelligence (O’Shea and Fieo, 2015) and executive attentional control (Kahneman, 1973). Moreover, an explanation in terms of attention and working memory capacity can explain individual differences in perceiving audiovisual stimuli.

As a counterpart to inattentional blindness, Macdonald and Lavie reported that people could also miss sounds in high-visual-load condition; a phenomenon which they described as “inattentional deafness” (Macdonald and Lavie, 2011). It stands in parallel with inattentional blindness, following the same procedure of reducing perceptual processing of task-irrelevant information in high-load tasks. Therefore, one could expect various forms of “inattentional deafness” resembling the known forms of “inattentional blindness” (Mack and Rock, 1998), ranging from failing to recognize meaningful distractor objects (Lavie et al., 2009) to failing to notice the presence of stimuli (Neisser and Becklen, 1975).

Earlier research has also shown the benefit of vision in speech-reception (Musacchia et al., 2007). By contrast, it has also been shown that in situations of uncertainty, observers tend to follow the more reliable auditory cue (Apthorp et al., 2013). Very mild forms or hearing damage might lead to reduced speech intelligibility (Bharadwaj et al., 2014; Füllgrabe et al., 2015) and thus a stronger reliance on visual cues. But, it was also observed that some persons are simply more auditory dominated while others are more visual dominated (Giard and Peronnet, 1999).

The above discussion indicates that there might be individual differences in the way people perceive audiovisual stimuli that would be more pronounced in a rather complicated audiovisual environment, possibly due to individual differences in distractibility. Individual levels of distractibility can vary from slight facilitation from a noisy background to severe disruption (Ellermeier and Zimmer, 1997). It has been suggested that individual differences in working memory capacity underlie individual differences in susceptibility to auditory distraction in most tasks and contexts (Sörqvist and Rönnberg, 2014). The findings on working memory capacity reflect individual differences in the ability to control attention and avoid distraction (Conway et al., 2001). It has been shown that high-working memory capacity individuals are less susceptible to the effects of auditory distractors (Beaman, 2004; Sörqvist, 2010). A recent study showed that attention restoration is achieved through increased exposure to natural sounds, while conversely, human-caused sounds reduce attention restoration (Abbott et al., 2016).

Throughout this article, the personal factor which was discussed above and that is expected to influence how persons perceive and appraise a combined auditive and visual stimulus will be labeled *audiovisual aptitude*. The term *aptitude* was chosen to highlight our hypothesis that this personal factor reflects a natural ability to process audiovisual scenes. This ability includes focusing on either (the visual or auditory) part of the scene and its composition in both simple and complex scenes. Its detailed meaning will further be explored in the discussion section.

This paper uses an audiovisual deviant detection experiment, with real-life scenes containing multiple visual and audio elements, to categorize persons according to their auditory acuity and their distractibility by incongruent visual stimuli. Two previously conducted experiments (labeled experiments 2 and 3 in the following sections) have been reanalyzed by including audiovisual aptitude as a personal factor. Audiovisual aptitude is expected to modify the effect of the view from the window on reported noise annoyance in Experiment 2. In Experiment 3, it modifies the effect of sonic and visual stimuli on pleasantness of walking across a bridge.

The audiovisual deviant detection experiment was designed to focus on the skills and sensitivities that matter for environmental sound perception. Previous research has shown that sounds that can be recognized relate to the overall appraisal of soundscapes in public places such as parks (Pilcher et al., 2009; Axelsson et al., 2010; Miller et al., 2018). Likewise, it was shown that noticing sounds from outside influences annoyance at home (De Coensel et al., 2009). In general, perception is a comprehensive process, in which a single factor sometimes cannot explain the final result (Botteldooren et al., 2006; Brown, 2012). Thus, the first part was designed to test the participant’s ability to analyze complex auditory scenes and identify individual sounds in it. An ecologically valid setting assures that participants can also rely on personal experience and context-related expectation, factors that will also influence the appraisal of the environment in everyday life. A deviant detection task is chosen where the deviant is a complex auditory scene in which one sound is missing. To explore the influence of visual information on sound perception that is explained above, the second part of the test adds the visual context that matches the auditory scene. Congruent visual information on the deviant (missing sound) would be beneficial in general for the deviant detection task. Yet, as people are in general expected to be more visually guided (Colavita effect), participants could then simply detect the visual deviant, which would not be very instructive for identifying their audiovisual aptitude. Hence, the information on the deviant was made incongruent between the visual and the auditory information, making distraction and perceptual load dominant mechanisms.

## Methodology

### Overview

This study uses three experiments conducted by the same participants to identify the personal differences in audiovisual aptitude (Experiment 1) and to explore how these differences influence perception of the environment (Experiments 2 and 3).

The first experiment explores audiovisual aptitude. It consists of a blind audio test (Part 1) and audiovisual test (Part 2) sharing the same audio track. During both tests, participants were requested to detect the deviant auditory stimulus amongst three fragments. This experiment contained four scenarios, in which either the audio or visuals altered. This ecologically valid alternative to simple psychological stimuli is intended to investigate whether a person’s visual attention mechanism dominates auditory attention.

Meanwhile, the same participants joined the other two experiments, one focusing on road traffic annoyance at home and the other on the perceived quality of the public space. These have been analyzed in view of the audiovisual aptitude. This setting allows to explore whether the personal audiovisual aptitude identified in Experiment 1 can be used to explain differences in response in the other two experiments.

With the criteria of good (peripheral) hearing and completing the whole experiment, this study collected 68 participants (28 Female, *M*_age_ = 27.9, *SD* = 5.05, range: 20–46 years, 48 obtained a master degree or higher). In later analysis, participants were classified based on gender, age (divided into two groups by median value 27, group 1: 20–27 years, 37 participants, *M*_age_ = 24.2, *SD* = 1.8; group 2: 31 participants, 28–46 years, *M*_age_ = 32.5, *SD* = 3.9) and education. All the principles outlined in the Helsinki Declaration of 1975, as revised in 2000 (World Medical Association, 2001), have been followed in all the experiments involving human subjects. All participants signed an informed consent form before the start of the experiments.

### Experiment 1: Audiovisual Aptitude

#### Layout of the Paired Test

As shown in **Table 1**, the audio test (Part 1) only contains the audio content, while the video test (Part 2) contains both sound and vision. In each part, participants were asked a single question after experiencing the three items: ‘Which of the three items sounds most differently from the other two?’. In Part 1, item 2 was the correct answer, whereas in Part 2 item 5 was the correct answer. During the analysis stage, in Part 1, choosing item 2 will be marked as correct, and consequently, choosing item 1 or 3 will be considered as mistake 1 (M1). In Part 2, item 5 is correct, and 4 and 6 mistakes (M2).

**Table 1:** Overview of audio-visual scenarios studied in Experiment 1.

	Item Number	File format	Content		Mistake type
			Auditory	Vision	
Part 1	1	Audio	Background sound + AAO	Black screen	M1
	2	Audio	Background sound	Black screen	
	3	Audio	Background sound + AAO	Black screen	M1
Part 2	4	Video	Background sound + AAO^∗^	Background view + VAO^∗^	M2
	5	Video	Background sound	Background view + VAO	
	6	Video	Background sound + AAO	Background view	M2

*^∗^Congruent visual attention attracting object (VAO) and matching auditory attention attracting object (AAO).*

#### Scenarios Content

**FIGURE 1:**
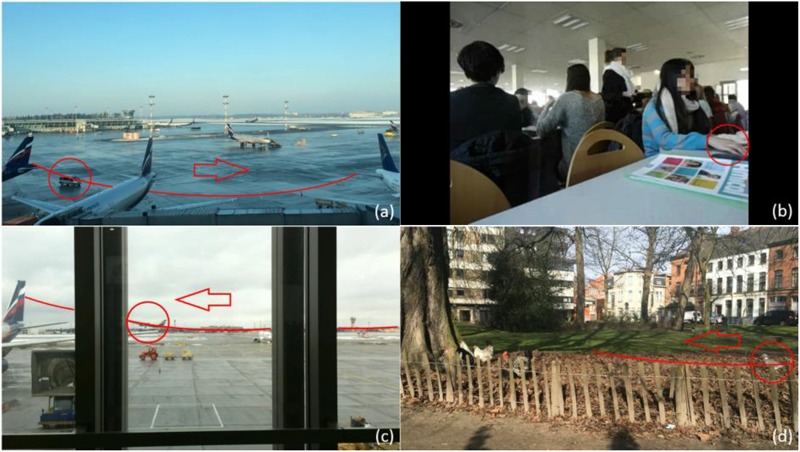
Snapshots for four scenarios: **(a)** airport car; **(b)** restaurant; **(c)** aircraft; and **(d)** city park.

This study uses four different scenarios. Content details of the videos are listed in **Table 2**. **Figure 1** shows screenshots of the four scenarios.

**Table 2:** Visual and auditory context for each of the scenarios used in the audiovisual aptitude experiment together with congruent visual attention attracting object (VAO) and matching auditory attention attracting object (AAO).

Number	a	b	c	d
Scenario	Airport car	Restaurant	Aircraft	City park
Main visual context (background view)	Terminal window view to parking apron	Student restaurant at sitting position	Terminal window view to airport runway	A bunch of chicken in the park
Main auditory context (background sound)	Broadcasting, people talking, aircraft engine	People talking, eating, forks and plates	Airport outside sound, wind, shuttle bus passing	Chicken crowing and walking on fallen leaves
VAO	Shuttle bus passing	Tapping finger	Departing aircraft	Walking pigeon
AAO	Shuttle bus sound	Finger tapping sound	Aircraft departing sound	Pigeon cooing, walking on leaves
Total duration	0:27	0:35	1:00	0:55
AO duration	0:12	0:12	0:24	0:11
(percentage)	(44.4%)	(34.3%)	(40%)	(20%)

In **Figure 1**, the object (VAO) that is absent in one of the videos in each scenario is indicated with a circle, while its path and moving direction are shown with the solid lines and arrows. Scenario (a) shows a view of a tarmac through a terminal window, with several aircrafts and a few shuttle busses far in the scene. The background sound consists of terminal announcements and people talking. Scenario (b) is a crowded student restaurant, with people eating, talking and laughing (forming the background sound). The attention attracting object in scenario (b) is a tapping finger, with its small movement within the range of the solid line circle as shown in **Figure 1b**. Scenario (c) shows an aircraft runway in front of a terminal window with many shuttle busses and vans moving around. Differently from scenario (a), the background of this scenario is an outdoor site with various mechanical sounds. The attention attracting object, a departing aircraft, occurs in the background of the scene. Scenario (d) shows a small city in a city outskirt, containing chickens on the left side of the screen, as well as a few cars passing by behind the park. The background sound here consists in chicken sounds, park sounds and city background sound. All four scenarios were recorded with a stable camera.

For each scenario, item 6 is the stimulus where the attracting object was removed from the visual. In scenario (a), (c), and (d), the (visually) attracting objects were removed. In scenario (b), the tapping finger was replaced by a stable hand lying on the table.

#### Procedure

This experiment was conducted scenario by scenario. In part 1 of the test, participants were asked to listen to items 1, 2, and 3 presented with audio only (black screen). In part 2, participants were asked to watch items 4, 5, and 6 from the same scenario. Once they finished a particular scenario, they could move on to the next one until all four scenarios were experienced.

The four scenarios were presented in random order and also the order of presenting the items was randomized. Each item could be played only once, and there was no backtrack and alteration once a single scenario was completed. All participant finished this experiment with the same headphones in the same quiet room (with a background noise of about 30 dBA).

In addition, personal information like age, gender and education level, as well as noise sensitivity [via Weinstein’s questionnaire (Weinstein, 1978)] were recorded (*M*_sensitivity_ = 79.40, *SD* = 10.95, participants were split into two groups with midpoint 73.5 afterwards). The hearing status of all participants was assessed via pure tone audiometry (PTA) carried out in a quiet but not sound-proof room using a regularly calibrated AC5Clinical Computer Audiometer.

### Experiment 2: Annoyance in Living Room

**FIGURE 2:**
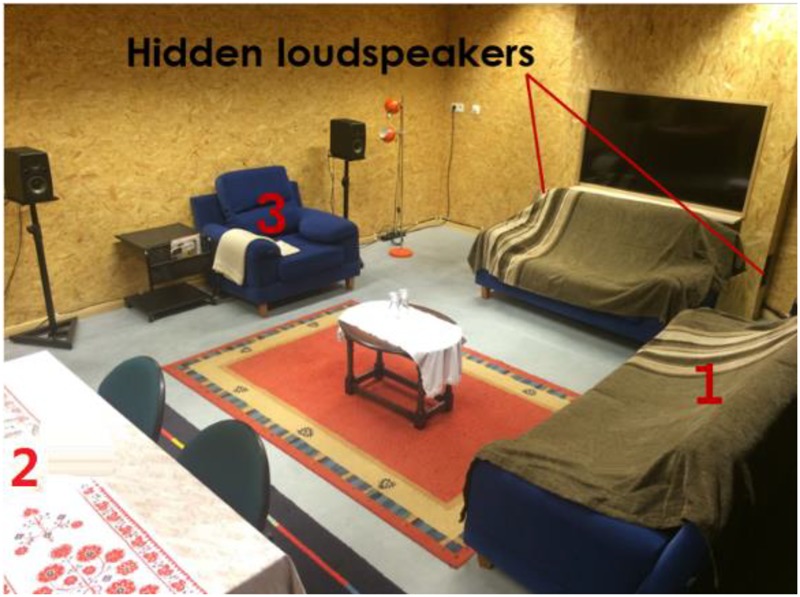
The mock-up living room with hidden loudspeakers indicated next to the mock-up window.

In a mock-up living room (**Figure 2**), participants were asked to engage in some light activities for 10 min while hearing highway traffic sounds. After 10 min, the standard ICBEN noise annoyance question was asked using an 11-point answering scale, referring to the past 10 min. This experiment was conducted with four sound pressure levels [45 dB(A), 50 dB(A), 55 dB(A), and 60 dB(A), measured in the center of the living room] corresponding to four different acoustical window insulation cases. The following 3 days, the same experimental procedure was repeated. However, while participants were led to believe that they simply evaluated again four window types, what actually changed was the video playing in the background to simulate a window view (**Table 3**). With this experimental design, we aimed to go beyond simple loudness evaluation (as can be expected by playing a short sound fragment only). In addition, we hid the true purpose, especially regarding our interest in the visuals displayed as a window view. More details on this experiment can be found in (Sun et al., 2018).

**Table 3:** Snapshots from the videos played in the mock-up window.

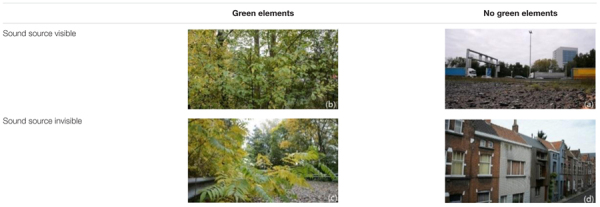

### Experiment 3: Perception of Public Space

**FIGURE 3:**
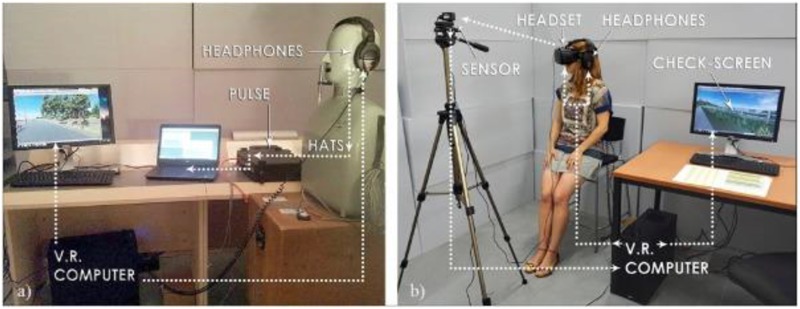
**(a)** Equipment used for calibration. **(b)** Equipment used for virtual reality experiment.

**FIGURE 4:**
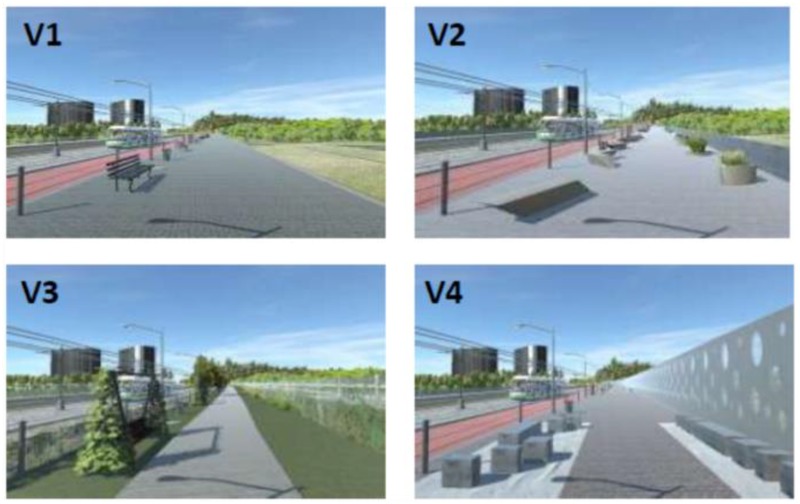
Snapshot of the virtual reality display of the four bridge designs; the barrier seen on the right progressively increases in height when going from V1 to V4, reducing the highway noise level.

The third experiment is complementary to the second one in two ways. Firstly, it considers the public space, more specifically the perceived environmental quality of a bridge crossing a ring road giving access to a park. Secondly, four visual designs were evaluated, hiding the fact that our interest is now in the effect of the noise coming from the highway below the bridge on audiovisual quality assessment. To achieve this, on each day of the experiment the participants evaluated a walk across the bridge in a virtual environment displayed to them using oculus rift (**Figure 3**). A sequence of four rather different visual designs were displayed to them each day (**Figure 4**), yet the sound coming from the highway under the bridge stayed the same. Participants were asked to rate the pleasantness of the total experience without specifically referring to sound. On the subsequent days, they evaluated visually identical environments yet the sound changed without informing the participants. More details on this experiment can be found in (Echevarria Sanchez et al., 2017).

In this experiment, participants were virtually moving across the bridge following a pre-defined path, but they could freely move their head. An important and interesting aspect that could be analyzed with this setup is the head movement, which is a proxy for their looking behavior, reflecting where people’s (visual) attention is directed to (Gibson and Pick, 1963). Recording the looking behavior allows assessing the frequency and total duration of gazing at the highway during the walk. This counting is based on the head movement of the participants and the screen middle point is used as a proxy for the visual focus point. This recording in only performed with the four matching situations (visual designs with the corresponding sonic environments).

### Statistical Analysis

To test whether the personal factors have an impact on the results of part 1 and 2 in Experiment 1, a repeated analysis of variance (anova) test was conducted. To observe the relation between a sound factor (the duration of the attention attracting object) and the overall result of part 1 and disparity between overall results in part 1 and 2, a linear regression was performed. Furthermore, in Experiments 2 and 3, first, a generalized linear model is built to find the fittest classification of participants through Experiment 1 – that is the classification that results in the best model quality. Then, a mixed-effect generalized linear model targeting at noise annoyance (Experiment 2) and pleasantness (Experiment 3) is conducted, using ‘participant’ as a random factor to generalize the results, accounting for various factors including the fittest personal factor via Experiment 1. The Akaike Information Criterion (AIC) is used to rate the model quality (models with smaller AIC values fit better). At last, an anova test is conducted to check the impact of personal factors on the gazing time in Experiment 3. The statistics analysis in this study was conducted in SPSS statistics (version 25).

## Results and Analysis

### Audiovisual Aptitude

#### Overview

**FIGURE 5:**
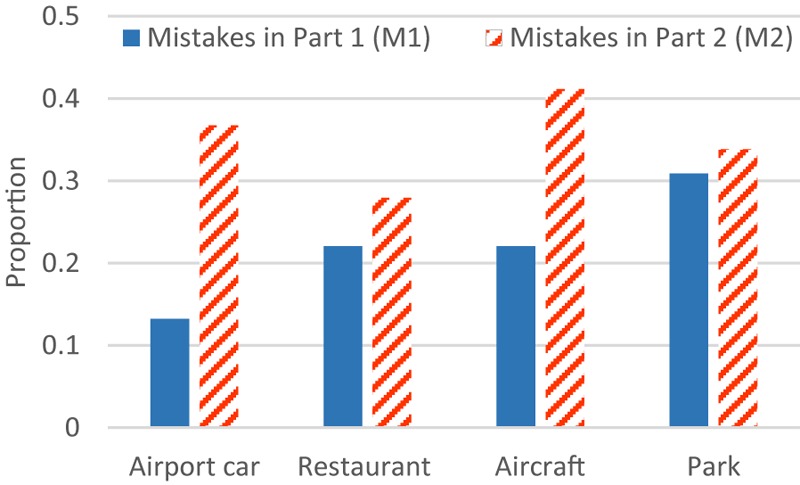
Proportion of the participants making mistakes in different scenarios of the aptitude experiment.

**Figure 5** shows the percentage of the participants that made a mistake in different parts of the audiovisual aptitude experiment. In part 1 (M1), scenario ‘park’ is where people made most mistakes while scenario ‘airport car’ led to the smallest number of mistakes. Despite the scenario differences, task performance in general decreases by adding a visual setting containing incongruent information on the deviant. Comparing the differences between M1 and M2, visual information makes the task performance significantly worse in some scenarios (‘airport car’ and ‘aircraft’), while in other scenarios, it has less effect. Further analysis will focus on personal factors that can be deduced.

#### Effect of Personal Factor

**FIGURE 6:**
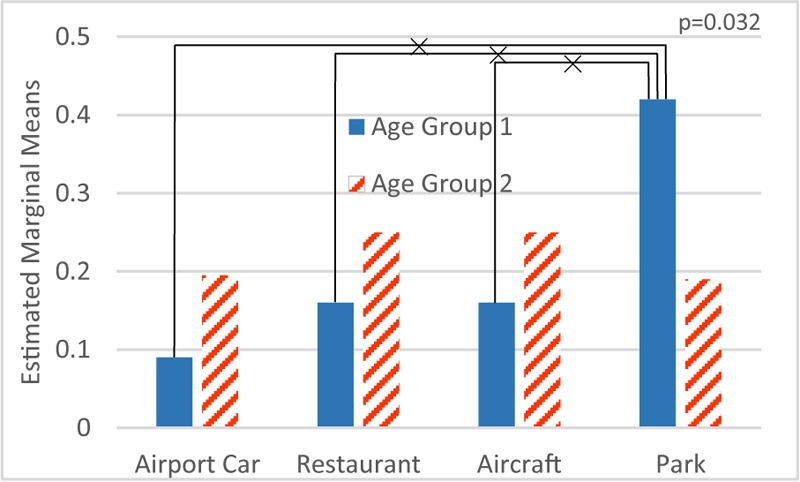
Interaction between scenario and age on M1 mistakes (Age Group 2 is older than Age Group 1). ×: population marginal means significantly different.

Aiming at M1, an anova test with factor scenario and various personal factors was made. The result shows that the factor education (*F*_1,264_ = 2.31; *p* > 0.05), gender (*F*_1,264_ = 1.25; *p* > 0.05), noise sensitivity (*F*_1,264_ = 0.052; *p* > 0.05) and age (*F*_1,264_ = 0.11; *p* > 0.05) are not significant. Interestingly, the interaction between the factors scenario and age is significant (*F*_3,264_ = 2.97; *p* < 0.05), as shown in **Figure 6**.

**FIGURE 7:**
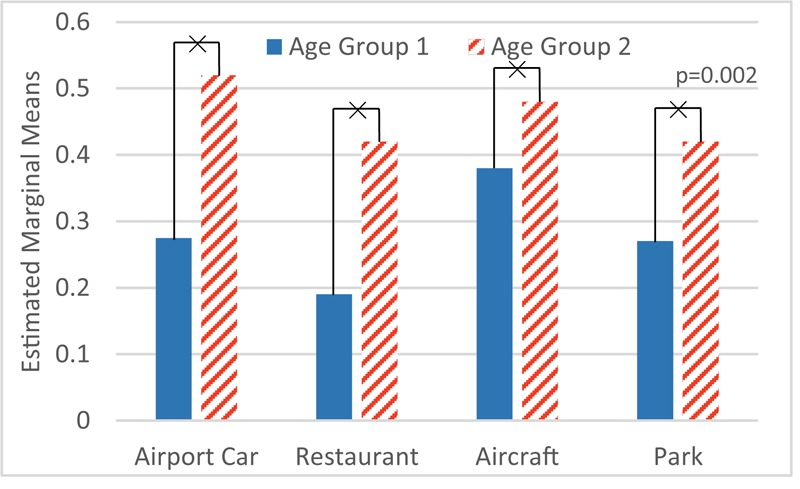
Age effect on M2 mistakes. ×: population marginal means significantly different.

On the other hand, the same procedure applied to M2 reveals that the factors education (*F*_1,264_ = 1.11; *p* > 0.05), gender (*F*_1,264_ = 0.46; *p* > 0.05) and noise sensitivity (*F*_1,264_ = 0.054; *p* > 0.05) are not significant, while age (*F*_1,264_ = 9.98; *p* < 0.01) is a significant factor, as shown in **Figure 7**.

As can be seen in part 1, factor age itself has no statistical significance on M1. Still there is a very strong interaction between age and scenario. Younger participants made more errors in scenario ‘park’ (**Figure 6**). In part 2 of the experiment, age is a statistically significant factor, namely older participants made more mistakes than younger ones in all scenarios (**Figure 7**).

**FIGURE 8:**
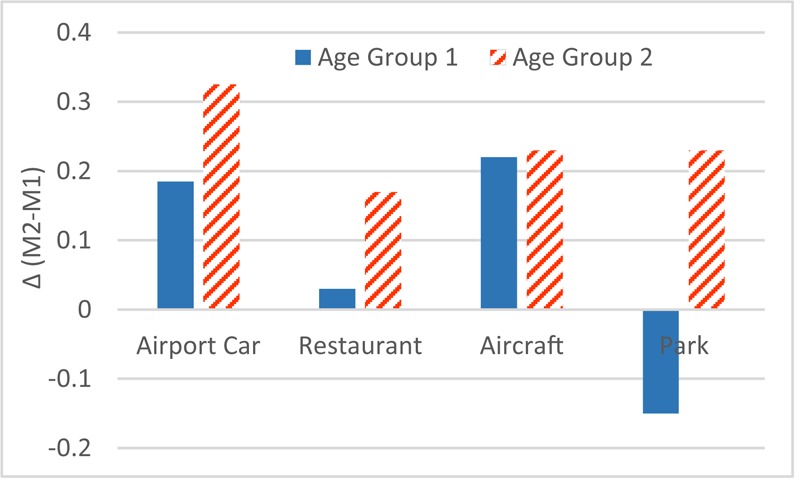
Disparity of M1 and M2 by age groups.

Furthermore, **Figure 8** shows the difference between results in part 1 and part 2, which suggests the effect of visual distraction on each age group in the four scenarios. A rather smaller variation among all four scenarios occurs in older participants.

#### Effect of Sound Features

**FIGURE 9:**
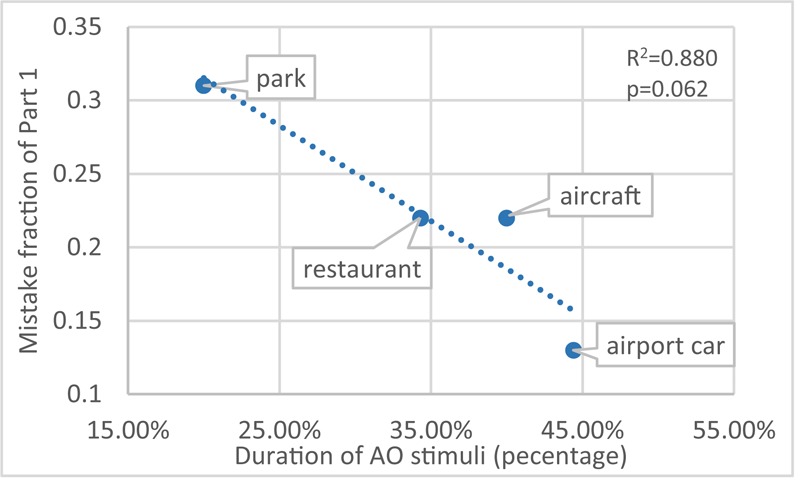
Correlation between duration (%) of AO stimuli and M1.

The observation task in part 1 could be described as a pure sound deviant detection. The variation of results between each scenario (M1, **Figure 5**) should be ascribed to the sound itself. One feature that differs between scenarios is the total duration (%) of the attracting object (AO) stimuli, as shown in **Table 2**. A one-way anova test involving duration (%) as a factor on the results of M1 (on each participant) shows it has statistical significance (*F*_3,264_ = 2.54; *p* < 0.05). In **Figure 9**, the correlation between AO duration (%) and M1 also supports the hypothesis that longer AO duration (%) decreases the difficulty of the sonic deviant detection task; the chance of making errors increases with decreasing duration.

**FIGURE 10:**
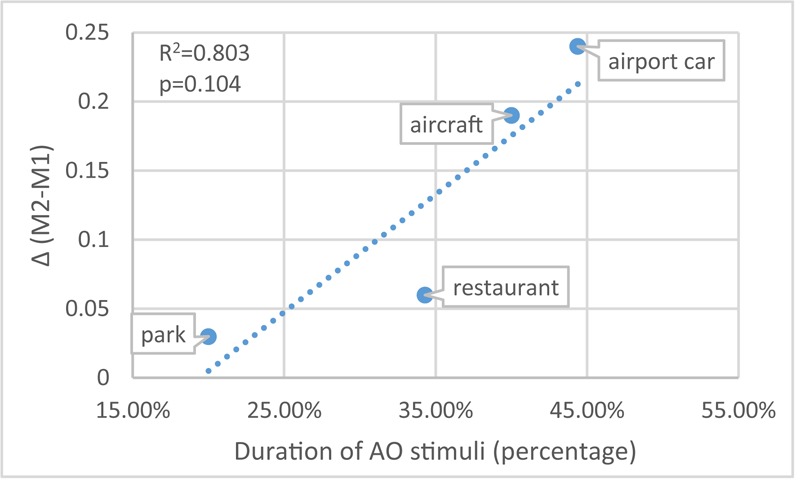
The correlation between AO duration (%) and Δ (M2-M1) (disparity of M1 and M2).

**FIGURE 11:**
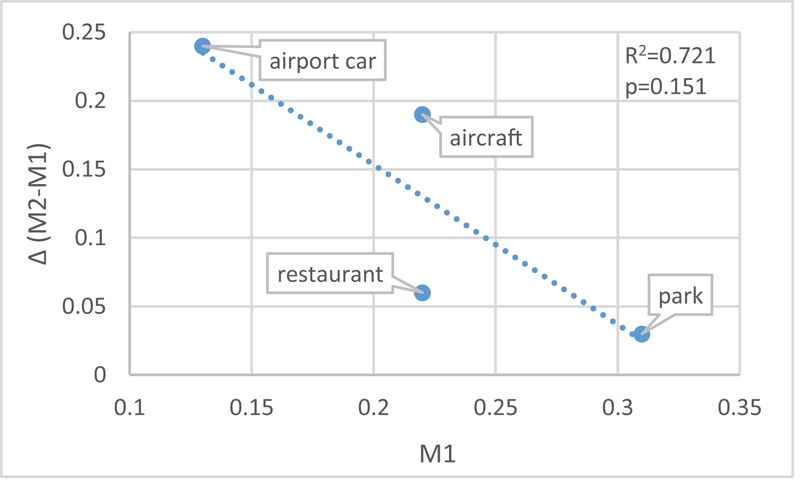
The correlation between M1 and Δ (M2-M1).

In **Figure 5**, the difference between M1 and M2 suggests that the mistakes caused by the incongruent visual information also span a wide range: scenario ‘airport car’ has the biggest [Δ(M2 - M1) = 0.24] and scenario ‘park’ has the smallest (Δ = 0.03) effect. This trend (**Figure 10**) also applies to the other two scenarios – scenario ‘aircraft’ (duration of AO = 40%; Δ = 0.19) and scenario ‘restaurant’ (duration of AO = 34.3%; Δ = 0.06). Despite the correlation between the duration (%) of AO and M1 (**Figure 9**). **Figure 11** further shows the correlation between M1 and Δ.

#### Clustering by Audiovisual Aptitude

**FIGURE 12:**
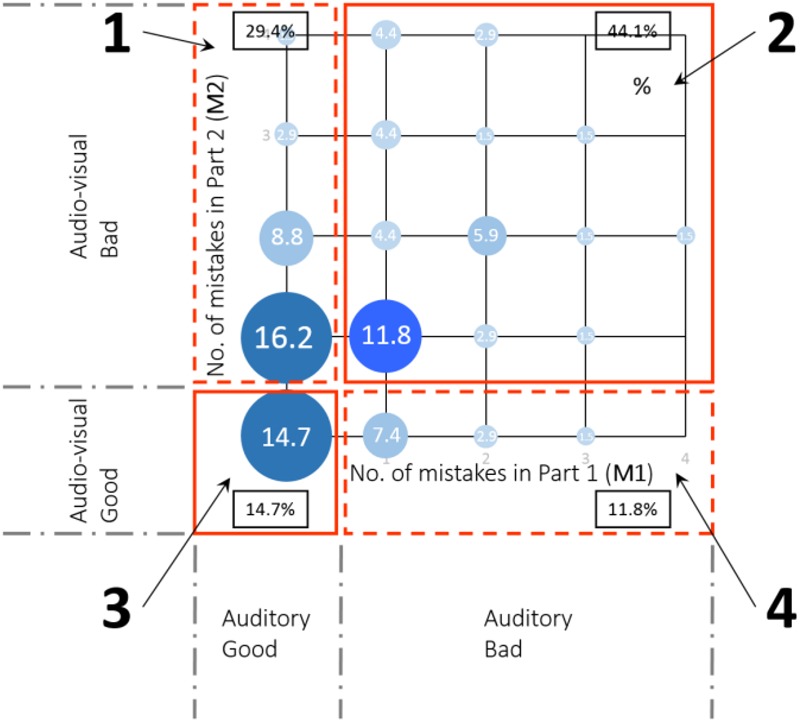
Participants grouping in the audiovisual aptitude experiment.

Combining the results of part 1 and part 2 in two dimensions (**Figure 12**) gives a clear view of the distribution of the participants. Participants were categorized into four groups. Group 1 (29.4%) are participants who made no mistakes in Part 1 but made at least one mistake after introducing the visual information (Part 2). Participants in group 2 (44.1%) made at least one mistake in both tests. On the contrary, group 3 (14.7%) are participants who made no mistake in any of the tests. Participants in group 4 (11.8%) made at least one mistake in Part 1, but flawlessly performed after introducing the visual information (Part 2).

These four groups generally represent different reactions toward the audiovisual stimuli, which would affect the perception as in the task performance. In the following analysis of the second and third experiment, this classification of participants will be referred to as audiovisual aptitude.

### Effect of Audiovisual Aptitude on Annoyance at Home

Previous analysis of this experiment showed the dominating effect of the sound level on noise annoyance and a smaller influence of the window view (Sun et al., 2018). To test the effect of audiovisual aptitude, a generalized linear model was built targeting annoyance and involving only sound pressure levels and various ways of categorizing the four groups that were identified before. **Table 4** shows the comparison of models with different groupings, aiming at searching for the best model (with lowest information criterion). Model 14 is better than other models, even though it increases the degrees of freedom. More factors and interactions are included to model 14 using a stepwise adding/removing methodology. Statistical significance of model deviance reduction when including an additional variable has been checked by likelihood ratio testing (based on the Chi-square distribution). **Table 5** shows details of the best model (model 14+) with all statistically significant factors.

**Table 4:** Comparison between models in living room experiment.

Model	Aptitude clustering				df	Information criterion (Akaike corrected)
	1	2	3	4		
1	A	B	B	B	4	3961.255
2	B	A	B	B	4	3964.488
3	B	B	A	B	4	3961.430
4	B	B	B	A	4	3989.188
5	A	A	B	B	4	3990.073
6	A	B	A	B	4	3989.473
7	A	B	B	A	4	3988.186
8	A	A	B	C	5	3960.111
9	A	B	A	C	5	3987.032
10	A	B	C	A	5	4014.913
11	A	B	B	C	5	3991.336
12	A	B	C	B	5	3960.627
13	A	B	C	C	5	3991.185
14	A	B	C	D	6	3957.773
14+						3934.948

**Table 5:** Details of model 14+ in living room experiment.

Fixed effects	Target: annoyance at home			
Source	*F*	df1	df2	Sig.
Intercept	58.739	13	1.073	0.000
Noise sensitivity	6.663	1	1.073	0.010
SPL	242.440	3	1.073	0.000
Noise sensitivity^∗^Sound source	6.003	2	1.073	0.003
Audiovisual aptitude^∗^Green	2.451	7	1.073	0.017

*^∗^‘Participant’ is used as random factor.*

**FIGURE 13:**
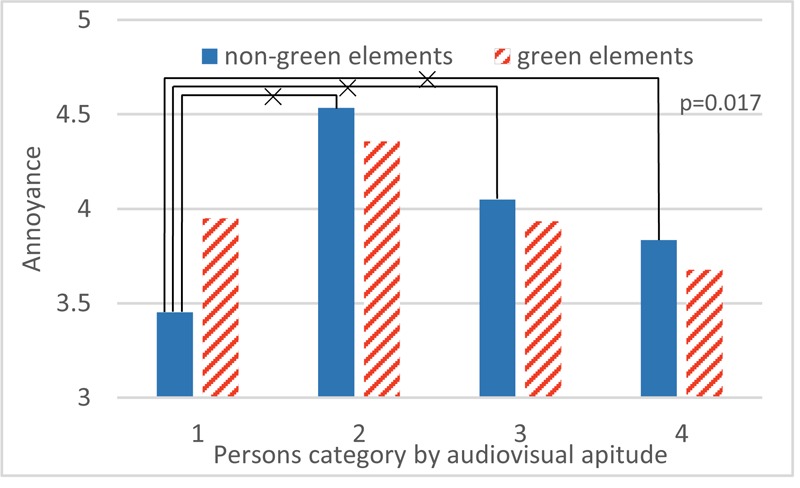
The interaction between audiovisual aptitude and green elements visibility on annoyance. ×: population marginal means significantly different.

Even though audiovisual aptitude is not significant as a single effect due to the presence of more important factors (namely SPL and noise sensitivity), there is a strong interaction between audiovisual aptitude and visibility of green elements (see the window scenes of the living room, section “Experiment 2: Annoyance in Living Room”). Details of this interaction are shown in **Figure 13**. Persons from all aptitude groups are slightly less annoyed when green elements are visible from the windows except in group 1. On the contrary, these persons that score very well on the purely auditory deviant detection task (Part 1, Experiment 1), but fail when an incongruent visual element is added (Part 2, Experiment 1), are less annoyed when a window scene without green elements is present.

### Effect of Audiovisual Aptitude on Perceived Quality of the Public Space

#### Models for Perceived Quality

Analysis of the third experiment showed the strong effect of the visual bridge design and a more moderate effect of highway sound on the pleasantness rating (Echevarria Sanchez et al., 2017). In this it should be noted that sound was only changed in between days to deliberately hide changes. The same procedure as in the previous experiment is applied, using a generalized linear model now targeting pleasantness and involving only sound environment, bridge design, and audiovisual aptitude. As in the previous experiment, statistical significance of model deviance reduction has been checked by likelihood ratio testing. Model 14+ adding more interactions to model 14 using subsequent adding and removing of factors, further improved the model quality. Details are shown in **Tables 6, 7**.

**Table 6:** Comparison between models in public space experiment.

Model	Aptitude clustering				df	Information criterion (Akaike corrected)
	1	2	3	4		
1	A	B	B	B	7	4161.258
2	B	A	B	B	7	4134.640
3	B	B	A	B	7	4160.538
4	B	B	B	A	7	4160.429
5	A	A	B	B	7	4161.331
6	A	B	A	B	7	4161.570
7	A	B	B	A	7	4161.065
8	A	A	B	C	8	4160.176
9	A	B	A	C	8	4164.030
10	A	B	C	A	8	4160.841
11	A	B	B	C	8	4213.013
12	A	B	C	B	8	4160.962
13	A	B	C	C	8	4161.575
14	A	B	C	D	9	4133.550
14+						4123.957

**Table 7:** Details of model 14+ in public space experiment.

Fixed effects	Target: pleasantness in public space			
Source	*F*	df1	df2	Sig.
Intercept	12.582	27	1.060	0.000
Bridge design	63.038	3	1.060	0.000
Sound environment	2.670	3	1.060	0.046
Audiovisual aptitude^∗^Bridge design	2.516	9	1.060	0.007
Audiovisual aptitude^∗^Sound environment	2.502	9	1.060	0.008

*^∗^‘Participant’ is used as random factor*.

**FIGURE 14:**
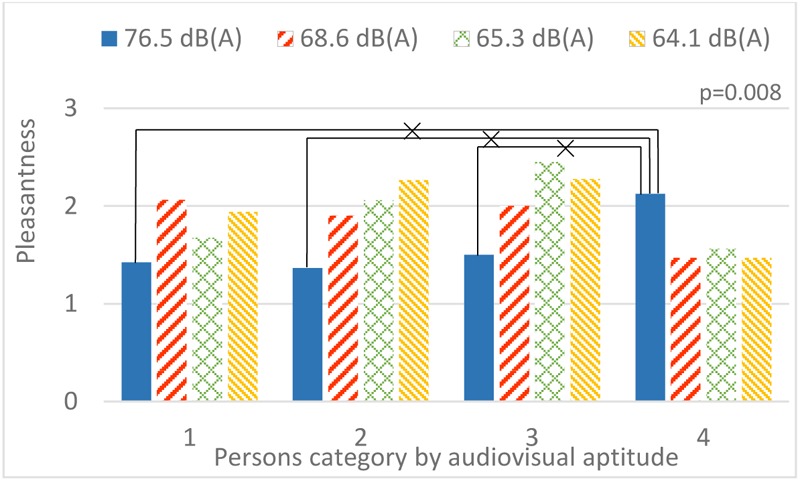
The interaction between audiovisual aptitude and sound environment (highway SPL is used as a label) on pleasantness. ×: population marginal means significantly different.

**FIGURE 15:**
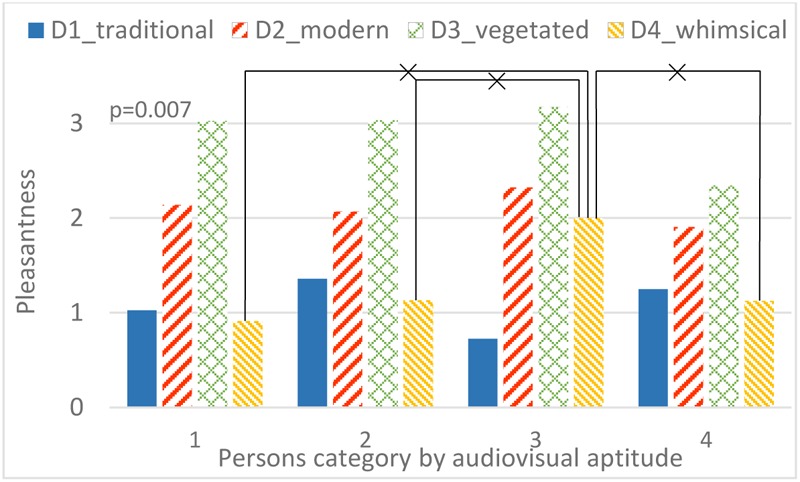
The interaction between audiovisual aptitude and bridge design on pleasantness. ×: population marginal means significantly different.

**FIGURE 16:**
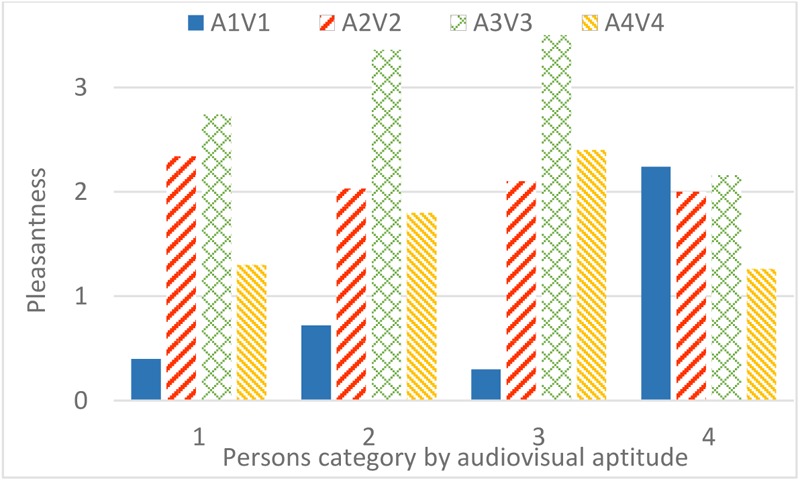
Effect of audiovisual aptitude on pleasantness of matching audiovisual designs.

A strong interaction occurs between audiovisual aptitude and both bridge design and sound environment. In **Figure 14**, only people from aptitude group 2 have an increasing pleasantness rating with lower contribution of highway sound. Group 1 and 3 have a special preference for the sound environment with the 2nd and 3rd strongest contribution of highway sound, 68.6 dB(A) and 65.3 dB(A), respectively. Oddly, people from group 4 prefer the sound environment with the strongest highway sound more than any others. In **Figure 15**, people in all aptitude groups show a common high appraisal of bridge design 3 (including vegetation, **Figure 4**, V3), followed by design 2. Designs 1 and 4 lead to relatively low pleasantness ratings, with design 4 being even slightly worse than design 1 for most people. However, the only exception is group 3 (those who performed without errors in the aptitude experiment, in both parts 1 and 2): design 4 is much higher rated than design 1. In addition, **Figure 16** shows the effect of audiovisual aptitude on pleasantness of the matching audiovisual combinations, namely the bridge design with the corresponding sonic environment. Persons from group 1, 2, and 3 share the similar trend, except for people from group 3 slightly preferring bridge 4 rather than bridge 2. However, for persons in group 4, bridge 4 is clearly the worst and the other three bridges do not differ from each other very much.

#### Looking Behavior Study: The Gazing Time

A one-way anova test with factor bridge design and gazing time (total time, **Table 8**) shows this is a statistical significant factor (*F*_3,224_ = 8.84; *p* < 0.01). It reveals that at bridges 1 and 2 (**Figure 4**, V1 and V2), people tend to look more often and longer at the highway. These two bridges both contain rather low edge barriers, visually exposing the sound source directly. Also, in all four bridge designs, the average gazing time is longer than the median gazing time, which shows that participants who actually look at the highway traffic do this for a longer time.

**Table 8:** Total gazing time for each bridge design.

Bridge designs	Gazing time					
	Total time (seconds)		Number of times		Average time (seconds)	
	Average	Median	Average	Median	Average	Median
1	14.58	11.9	2.84	3	4.85	4
2	14.48	11.6	2.88	3	4.50	4.06
3	7.81	4.6	1.72	1	2.97	3.05
4	7.19	5.7	1.53	1	3.83	2.95

An anova test targeting at total gazing time involving the factor bridge design and personal factors shows that education (*F*_1,220_ = 3.03; *p* > 0.05), gender (*F*_1,220_ = 2.50; *p* > 0.05), age (*F*_1,220_ = 3.77; *p* > 0.05), and noise sensitivity (*F*_1,220_ = 0.04; *p* > 0.05) have no statistical significance, while audiovisual aptitude (*F*_3,212_ = 2.73; *p* < 0.05) is significant. However, there is no strong interaction between the factors bridge design and audiovisual aptitude (*F*_9,212_ = 0.72; *p* > 0.05). Moreover, looking back at the overall pleasantness, no clear correlation between total gazing time and pleasantness is found (*F*_113,228_ = 0.64; *p* > 0.05).

**FIGURE 17:**
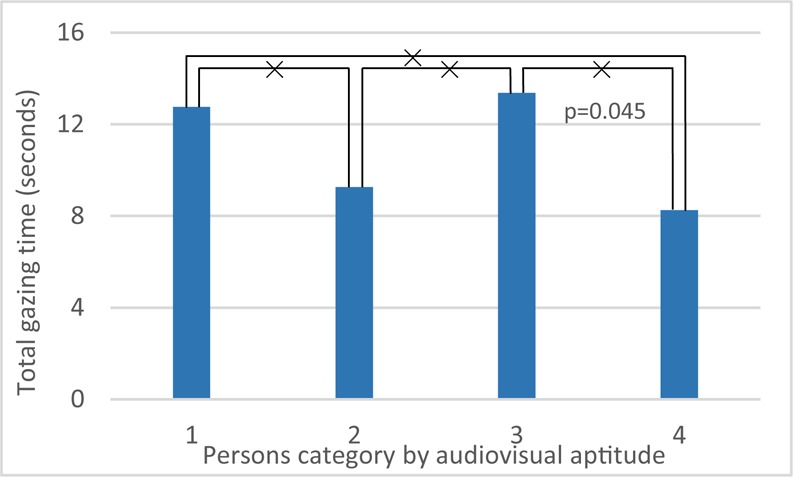
Effects of audiovisual aptitude on total gazing time. ×: population marginal means significantly different.

**FIGURE 18:**
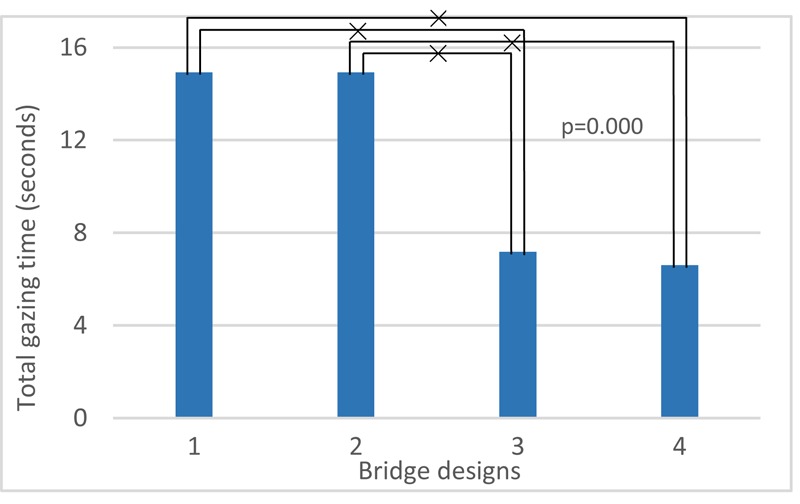
Effects of bridge designs on total gazing time. ×: population marginal means significantly different.

Note that in this section, the four bridges not only differ from each other by visual design, but also the sound level from the highway is decreasing from bridge 1 (highest) to bridge 4 (lowest). **Figure 17** shows that persons in aptitude groups 1 and 3, who made no errors in Part 1 of audiovisual aptitude experiment (Experiment 1), look at traffic longer than the other two groups. **Figure 18** shows that bridge 1 and 2, which have a rather low barrier and thus higher highway noise levels, result in more gazing time than in case of the other two bridges.

## Discussion

The goal of current study was to provide evidence for the existence of a personal factor that could influence the perception of landscape and soundscape and their interaction. For this purpose, an experiment (Experiment 1) was designed to explore the individual difference in capability for unraveling audiovisual stimuli and its distractibility from auditory acuity. This personal factor was labeled *audiovisual aptitude*. Two other experiments (Experiments 2 and 3) were re-analyzed involving this personal factor. We found that in Experiment 2, this individual difference modified the impact of window views on self-report noise annoyance in a living room context. In Experiment 3, this individual difference altered the impact of highway sound pressure level and visual bridge design on the pleasantness rating of a public space. It also affected the looking behavior during the perception of the public space.

Our audiovisual aptitude test categorizes people according to their ability to perform the purely auditory test at one hand and the audiovisual test at the other. It is a rather strict way of grouping participants in four groups. For instance, aptitude group 3 does not allow a single mistake. Each of the groups identified in **Figure 12** can be characterized in more detail and the underlying reasons for people to belong to this group may be explored. This also makes the definition of the factor audiovisual aptitude more precise.

For persons in aptitude group 1, incongruent visual information interferences the performance on the auditory task for the average person. They perform very well on the blind auditory test but start making mistakes once incongruent visual information is presented to them simultaneously. Macdonald and Lavie highlighted the level of perceptual load in a visual task as a critical determinant of inattentional deafness, an equivalent of inattentional blindness (Macdonald and Lavie, 2011). Persons in this group were successful in the sound deviant task with a low visual perceptual load (black screen, Part 1), but failed when the visual perceptual load increased (Part 2) which could be explained by being more vulnerable to inattentional deafness. Collignon et al. (2008) suggested the possibility of visual dominance in emotional processing under incongruent auditory and visual stimuli. However, this visual dominance in affect perception does not occur in a rigid manner, namely the visual dominance will disappear if the reliability of the visual stimuli is diminished (Collignon et al., 2008). The reliability of visual and auditory information influences the cross-modal asymmetry effects in temporal perception (Wada et al., 2003).

Group 2 contains most of the participants in this study. Although they often detect deviant auditory stimuli correctly with or without visual information, they make at least one error in both tasks with a slight tendency of making more errors when visual incongruent information is present (**Figure 12**). The complexity of the test arises either from the cocktail party effect (Conway et al., 2001) or the visual distraction effect on perception (Simons and Chabris, 1999). Both phenomena have been identified before. Hearing damage, even at a level where people would not report hearing problems or tonal audiometry does not show significant threshold shifts, could still cause reduced auditory scene analysis capacity (Füllgrabe et al., 2015). Auditory neuropathy has recently been identified as one possible cause (Bharadwaj et al., 2014). Although the age of the participants in this study does not warrant expecting a high incidence of hearing damage, some participants could clearly have more difficulties in performing the test. Also at the cognitive level we can expect some groups to perform worse (Edwards, 2016).

Persons in group 3 succeed in detecting the deviant sound in each of the four situations regardless of the presence of incongruent visual information. They could be labeled hearing specialists and are probably auditory dominated. Noise sensitivity was found before to be moderately stable and associated with current psychiatric disorder and a disposition to negative affectivity (Stansfeld, 1992), which is at least partly inherited (Heinonen-Guzejev, 2009). The present study included the Weinstein noise sensitivity survey. Persons in this group do not answer consistently different on this noise sensitivity questionnaire, which seems to indicate that another characteristic is measured by the proposed test. Other authors also noted that despite the fact that noise sensitivity has been established and widely applied in noise-related studies, it reveals only one personality trait. Miedema and Vos (2003) questioned the validity of ascribing noise sensitivity to a general negative affectivity among people. Recent research also showed that the personality had an independent effect on noise sensitivity (Shepherd et al., 2015).

Finally, group 4 contains people that seem to be helped by the incongruent visual information while detecting deviant sound environments. They are the smallest group in this study. For purely visual tasks, it was demonstrated that a single discrete visual distraction can improve the detectability of an unexpected object (Pammer et al., 2014). Yet, it is equally likely that the visual information gives them a clue on what sounds they need to listen for in the auditory deviant detection task. Some people may have acquired the skill to compensate for their inability to form auditory objects in an auditory scene analysis task via top down mechanisms grounded in visual information.

The usefulness of the personality factor identified by the proposed audiovisual test for understanding the perception of the soundscape, and specifically the interaction between the visual and the sonic environment in it, is illustrated with two experiments.

Experiment 2 focused on road traffic noise annoyance in a living room environment. Comparing predictive models showed that keeping the four groups identified above (as separate groups) explained the observations best. **Figure 13** further shows that participants belonging to aptitude groups 2, 3, and 4 reported less noise annoyance when green elements were visible from the window, which is consistent with many studies (Maffei et al., 2013; Van Renterghem and Botteldooren, 2016). However, persons belonging to group 1 behaved significantly differently. They reported more annoyance at the same noise exposure when green elements were shown in the window pane (**Table 3**). To explain these observations, it should first be noted that the green views in this case did not provide an appealing and readable green area following the reasoning in (Kaplan and Kaplan, 1989). Instead, it only served as a visual barrier between the window and a highway. For this reason, the positive effect found in other studies may be less pronounced or even reversed. The deviating influence of a green window view on the annoyance response in group 1 may be explained in several ways. Persons in this group were identified as visual dominant and the mediocre quality of the green may have a stronger negative effect on them. Such a green view is also incongruent with the sonic environment. Persons in aptitude group 1, which are easily distracted by incongruent visual information, may value congruence more and experience the expectation gap more strongly. This expectation gap could confuse them and push them to reporting more annoyance by the traffic noise.

The evaluation of the pleasantness of crossing a bridge over the highway using virtual reality (Experiment 3) also revealed significant differences between the audiovisual aptitude groups. **Figure 16** shows that the most obvious group with deviant pleasantness evaluation is group 4. These participants value the audiovisual design 1 (without barrier) much more than other participants and at the same time they seem to find less pleasure in the green design (A3V3). To investigate further the reasons for this deviant rating, a closer investigation of **Figures 14, 15** reveals that it is not the visibility of the source that makes the original situation (A1V1) more pleasurable but to some extent the higher highway noise level. However, the magnitude of the effect is much more pronounced in the physically matching situation. Thus, congruency of the audiovisual information seems to play a role. In the perceived restorativeness soundscape scale (PRSS) study, Payne pointed out that specific types of sounds and their associated meanings were more important in influencing the perceived restorativeness of the soundscape than its overall sound pressure level (Payne, 2013). Considering the relatively lower pleasantness rating of the green design (A3V3) in group 4 compared to the other groups, the effect in this case seems better explained by the lower pleasure rating of the visual design (D3) as seen in **Figure 15**. Combining all of these observations leads to the hypothesis that persons belonging to group 4 value congruency of audiovisual information and moreover prefer to see the highway that produces the sound they hear. This matches what could be expected by the description of possible traits within this group 4 given above: these people need visual information to understand the auditory scene. Not having this information leads to a lower pleasantness rating.

Also group 3 shows deviant pleasantness ratings, in particular they value the design including a high noise barrier (A4D4) more than others (**Figure 16**). Looking at **Figures 14, 15** it becomes clear that this is caused by a significantly higher pleasantness rating of visual design 4 even if averaged over combinations with different highway sound levels. Earlier, this group was identified as hearing specialists, persons that are very skillful in identifying deviant sounds and that do not get misled by incongruent visual information. At first sight, this may contradict the observation that the bridge design 4 is rated more pleasantly even if combined with different highway noise levels. However, the hypothesis is forwarded that seeing the high noise barrier already induces the feeling that highway noise will be mitigated, a fact that is highly appreciated by this group.

In addition, **Figure 14** shows that most participants (aptitude groups 1, 2, and 3) are following a trend of higher pleasantness rating with decreasing highway sound pressure level, despite the small difference between them. Even though the experiment was conducted on different days and the level difference can be as low as 1.2 dB(A), such a trend was still obtained. The presence of sounds that can create a frame of reference such as footsteps and a tram pass by could explain this (Echevarria Sanchez et al., 2017).

The virtual reality method used in Experiment 3 also allows to monitor the head movement of the participants in the study. Participants belonging to groups 1 and 3 turned their head significantly longer toward the cars on the highway. Participants in these groups make no errors on the auditory deviant detection task but may fail in the presence of incongruent visual information. Head movement is helpful in auditory scene analysis (Kondo et al., 2014), yet persons belonging to groups 1 and 3 are not expected to need this information as they are performing very well on the purely auditory test. A more plausible explanation for the observed difference between groups might be that it reflects a stronger focus on environmental sound.

Hence Experiments 2 and 3 show that the personal factor obtained from the aptitude experiment modifies perception of the audiovisual environment, both in a home setting and in the public space. This consistent and stable personal factor could be a potential modifier in studies on the interaction between visual and auditory information in perception experiments and could affect the way the urban environment is designed.

The core strength of the categorization should be ascribed to the aptitude experiment itself, so this experiment is analyzed in more detail. The test has been designed to assess the aptitude of participants in the auditory scene analysis step in auditory perception and to measure resistance against incongruent visual information. Indirectly it integrates an assessment of peripheral hearing status and attention focusing and gating capabilities of the person. For this reason, the test was based on ecologically valid and complex auditory and visual scenes rather than on more abstract test that are commonly used in psychology. This choice was made to maximize the probability of finding significant associations to the noise annoyance and public space perception. An appropriate test should be sensitive, reproducible, and easy to understand.

To guarantee sensitivity for all persons, the test consisted of four different contexts and deviants that could be more or less easily detected: then scenario ‘airport car’ would be the easiest one while scenario ‘park’ the hardest. This range in difficulty is mainly achieved by the duration (%) of AO stimuli as shown in Section “Effect of Sound Features.” **Figure 10** indicates that in scenario ‘airport car,’ the monitoring task is relatively easy (perceptual load of the task is low), the visual distraction is sufficiently working. While vice versa, in scenario ‘park,’ the monitoring task is rather hard (perceptual load of the task is high), the visual distractor processing tends to be less pronounced. This comparison agrees with perceptual load theory (Lavie, 1995). **Figure 11** confirms that the more difficult the purely auditory task, the lower the influence of the visual distractor.

Furthermore, the sensitivity of the test for age of the participant reflects the sensitivity of the test. Earlier research suggested that older adults were more affected by irrelevant speech in a monitoring task (Bell et al., 2008). The age deficits occurred in many conditions and increased with the similarity of distractor and target (Scialfa et al., 1998). Cohen and Gordon-Salant (2017) also stated that older adults may be more susceptible to irrelevant auditory and visual competition in a real-world environment. Some research has shown that older and younger persons obtained similar performance with purely auditory stimuli, but older adults have poor performance with audiovisual modality (Sommers et al., 2005). These findings are congruent with the presented study, as stated in Section “Effect of Personal Factor.” However, in part 1 of the audiovisual aptitude experiment, younger participants made less mistakes in all scenarios except for scenario ‘park’ (**Figure 6**). In **Figure 8**, the smaller variation in older participants suggests that the visual distraction tends to have a more equalized effect on them. However, for younger participants, there’s a bigger difference between scenarios, which might indicate that the visual distraction process highly depends on the context for younger people. Early research showed the effect of sound familiarity on recognition (Cycowicz and Friedman, 1998), which could suggest a large part of younger participants in this experiment were unfamiliar with a natural sonic environment.

The latter observation could lead to poor reproducibility of the test in another group of persons with different familiarity with the audiovisual scenes that are presented. This could be a plie for choosing a more abstract audiovisual test. The reported experiments were intended to show the existence of a difference in audiovisual aptitude between persons that could affect perception of the sonic and visual environment. It nevertheless has some limitations. An auditory deviant detection test with a limited number of scenarios will not reveal the full truth of above-mentioned hypothesis. The scenarios may not have been optimally chosen to balance familiarity with the environment amongst all participants. In addition to the age influence, other demographic factors may lead to a change in behavior in specific scenarios. For such an experiment, the number of participants matches widespread practice. However, using larger test populations may uncover other and more subtle influences and relationships. Also the verification – Experiments 2 and 3 – has certain shortcomings. In Section “Looking Behavior Study: The Gazing Time,” for instance, the head movement was used as a proxy for eye movement since no eye tracer, compatible with the VR headset, was available at the time of the experiment.

## Conclusion

Our study provides evidence for the existence of a personal factor that influences the effect of the view from a living room window on perceived noise annoyance by highway traffic noise and the effect of both the visual design and the highway noise level on perceived pleasantness of crossing a bridge over a highway. This personal factor, which we labeled audiovisual aptitude, may explain differences in perception of the (audiovisual) environment observed in other studies. It was shown that this personal factor differs from noise sensitivity, a known personality trait. It could become as important as noise sensitivity in understanding differences in perception of the living environment when both landscape and soundscape matter.

In this work, a deviant detection experiment was used to categorize persons according to their audiovisual aptitude. It was shown that categorization in four groups resulted in more performant models for predicting the above-mentioned influences than using less groups. Each group could be linked to personal factors identified previously in literature. Nevertheless, it can be expected that such an extensive test resulting in four groups might not be necessary. Based on the insights gained in this work, an audiovisual aptitude questionnaire may be constructed.

Future research may also focus on finding the neurological basis for the difference in audiovisual aptitude between persons. Recent research shows that high noise sensitivity is associated with altered sound feature encoding and attenuated discrimination of sound noisiness in the auditory cortex (Kliuchko et al., 2016). Audiovisual aptitude is expected to be related to attention moderated auditory scene analysis.

## Ethics Statement

This study was carried out in accordance with the recommendations of Good Clinical Practice (ICH/GCP), Commission for Medical Ethics [registration number BE670201628136 (31-03-2016)] with written informed consent from all subjects. All subjects gave written informed consent in accordance with the Declaration of Helsinki. The protocol was approved by the Commission for Medical Ethics.

## Author Contributions

KS and GES carried out the experiments under the supervision of BDC, TVR, and DB. KS performed the analytic calculations. KS took the lead to wrote the manuscript. All authors provided critical feedback and helped to shape the research, analysis and manuscript.

## Conflict of Interest Statement

The authors declare that the research was conducted in the absence of any commercial or financial relationships that could be construed as a potential conflict of interest.

## References

[B1] Abbott L. C. Taff B. D. Newman P. Benfield J. A. Mowen A. J. (2016). The influence of natural sounds on attention restoration. *J. Park Recreat. Admi.* 34 5–15. 10.18666/JPRA-2016-V34-I3-6893

[B2] Apthorp D. Alais D. Boenke L. T. (2013). Flash illusions induced by visual, auditory, and audiovisual stimuli. *J. Vis.* 13:3. 10.1167/13.5.3 23547105

[B3] Axelsson Ö. Nilsson M. E. Berglund B. (2010). A principal components model of soundscape perception. *J. Acoust. Soc. Am.* 128 2836–2846. 10.1121/1.3493436 21110579

[B4] Beaman C. P. (2004). The irrelevant sound phenomenon revisited: what role for working memory capacity? *J. Exp. Psychol. Learn. Mem. Cogn.* 30 1106–1118. 10.1037/0278-7393.30.5.1106 15355139

[B5] Bell R. Buchner A. Mund I. (2008). Age-related differences in irrelevant-speech effects. *Psychol. Aging* 23 377–391. 10.1037/0882-7974.23.2.377 18573011

[B6] Bharadwaj H. M. Verhulst S. Shaheen L. Liberman M. C. Shinn-Cunningham B. G. (2014). Cochlear neuropathy and the coding of supra-threshold sound. *Front. Syst. Neurosci.* 8:26 10.3389/fnsys.2014.00026 PMC3930880 24600357

[B7] Botteldooren D. Andringa T. Aspuru I. Brown A. L. Dubois D. Guastavino C. (2015). “From sonic environment to soundscape,” in *Soundscape and the Built Environment* Vol. 36 eds Jian K. Brigitte S. F. Boca R. (Boca Raton, FL: CRC Press) 17–42. 10.1201/b19145-3

[B8] Botteldooren D. De Coensel B. De Muer T. (2006). The temporal structure of urban soundscapes. *J. Sound Vib.* 292 105–123. 10.1016/j.jsv.2005.07.026

[B9] Brown A. L. (2012). A review of progress in soundscapes and an approach to soundscape planning. *Int. J. Acoust. Vib.* 17 73–81. 10.20855/ijav.2012.17.2302

[B10] Cartwright-Finch U. Lavie N. (2007). The role of perceptual load in inattentional blindness. *Cognition* 102 321–340. 10.1016/j.cognition.2006.01.002 16480973

[B11] Cohen J. I. Gordon-Salant S. (2017). The effect of visual distraction on auditory-visual speech perception by younger and older listeners. *J. Acoust. Soc. Am.* 141 EL470–EL476. 10.1121/1.4983399 28599569 PMC5724720

[B12] Colavita F. B. (1974). Human sensory dominance. *Atten. Percept. Psychophys.* 16 409–412. 10.3758/BF03203962

[B13] Collignon O. Girard S. Gosselin F. Roy S. Saint-Amour D. Lassonde M. (2008). Audio-visual integration of emotion expression. *Brain Res.* 1242 126–135. 10.1016/j.brainres.2008.04.023 18495094

[B14] Conway A. R. Cowan N. Bunting M. F. (2001). The cocktail party phenomenon revisited: the importance of working memory capacity. *Psychon. Bull. Rev.* 8 331–335. 10.3758/BF03196169 11495122

[B15] Cycowicz Y. M. Friedman D. (1998). Effect of sound familiarity on the event-related potentials elicited by novel environmental sounds. *Brain Cogn.* 36 30–51. 10.1006/brcg.1997.0955 9500881

[B16] De Coensel B. Botteldooren D. De Muer T. Berglund B. Nilsson M. E. Lercher P. (2009). A model for the perception of environmental sound based on notice-events. *J. Acoust. Soc. Am.* 126 656–665. 10.1121/1.3158601 19640031

[B17] Echevarria Sanchez G. M. Van Renterghem T. Sun K. De Coensel B. Botteldooren D. (2017). Using Virtual Reality for assessing the role of noise in the audio-visual design of an urban public space. *Landsc. Urban Plan.* 167 98–107. 10.1016/j.landurbplan.2017.05.018

[B18] Edwards B. (2016). A model of auditory-cognitive processing and relevance to clinical applicability. *Ear Hear.* 37 85S–91S. 10.1097/AUD.0000000000000308 27355775

[B19] Ellermeier W. Zimmer K. (1997). Individual differences in susceptibility to the “irrelevant speech effect”. *J. Acoust. Soc. Am.* 102 2191–2199. 10.1121/1.419596 9348677

[B20] Eriksen B. A. Eriksen C. W. (1974). Effects of noise letters upon the identification of a target letter in a nonsearch task. *Atten. Percept. Psychophys.* 16 143–149. 10.3758/BF03203267

[B21] Filipan K. Boes M. De Coensel B. Lavandier C. Delaitre P. Domitroviæ H. (2017). The personal viewpoint on the meaning of tranquility affects the appraisal of the urban park soundscape. *Appl. Sci.* 7:91 10.3390/app7010091

[B22] Fougnie D. Marois R. (2007). Executive working memory load induces inattentional blindness. *Psychon. Bull. Rev.* 14 142–147. 10.3758/BF03194041 17546744

[B23] Füllgrabe C. Moore B. C. Stone M. A. (2015). Age-group differences in speech identification despite matched audiometrically normal hearing: contributions from auditory temporal processing and cognition. *Front. Aging Neurosci.* 6:347. 10.3389/fnagi.2014.00347 25628563 PMC4292733

[B24] Giard M. H. Peronnet F. (1999). Auditory-visual integration during multimodal object recognition in humans: a behavioral and electrophysiological study. *J. Cogn. Neurosci.* 11 473–490. 10.1162/089892999563544 10511637

[B25] Gibson J. J. Pick A. D. (1963). Perception of another person’s looking behavior. *Am. J. Psychol.* 76 386–394. 10.2307/1419779 13947729

[B26] Graham E. R. Burke D. M. (2011). Aging increases inattentional blindness to the gorilla in our midst. *Psychol. Aging* 26 162–166. 10.1037/a0020647 21261412 PMC3062668

[B27] Heinonen-Guzejev M. (2009). *Noise Sensitivity Medical, Psychological and Genetic Aspects.* Doctoral dissertation, University of Helsinki Helsinki.

[B28] ISO (2014). *ISO 12913-1:2014 Acoustics — Soundscape — Part 1: Definition and Conceptual Framework.* Geneva: International Organization for Standardization.

[B29] Jiang Y. Chun M. M. (2001). Selective attention modulates implicit learning. *Q. J. Exp. Psychol. A* 54 1105–1124. 10.1080/713756001 11765735

[B30] Kahneman D. (1973). *Attention and Effort*, Vol. 1063 Englewood Cliffs, NJ: Prentice-Hall

[B31] Kaplan R. Kaplan S. (1989). *The Experience of Nature: A Psychological Perspective.* New York, NY: Cambridge University Press.

[B32] Kliuchko M. Heinonen-Guzejev M. Vuust P. Tervaniemi M. Brattico E. (2016). A window into the brain mechanisms associated with noise sensitivity. *Sci. Rep.* 6:39236. 10.1038/srep39236 27976708 PMC5157031

[B33] Kondo H. M. Toshima I. Pressnitzer D. Kashino M. (2014). Probing the time course of head-motion cues integration during auditory scene analysis. *Front. Neurosci.* 8:170. 10.3389/fnins.2014.00170 25009456 PMC4067593

[B34] Lavie N. (1995). Perceptual load as a necessary condition for selective attention. *J. Exp. Psychol. Hum. Percept. Perform.* 21 451–468. 10.1037/0096-1523.21.3.451 7790827

[B35] Lavie N. Fox E. (2000). The role of perceptual load in negative priming. *J. Exp. Psychol. Hum. Percept. Perform.* 26 1038–1052. 10.1037/0096-1523.26.3.1038 10884008

[B36] Lavie N. Lin Z. Zokaei N. Thoma V. (2009). The role of perceptual load in object recognition. *J. Exp. Psychol. Hum. Percept. Perform.* 35 1346–1358. 10.1037/a0016454 19803641 PMC2759815

[B37] Leung T. M. Xu J. M. Chau C. K. Tang S. K. (2017). The effects of neighborhood views containing multiple environmental features on road traffic noise perception at dwellings. *J. Acoust. Soc. Am.* 141 2399–2407. 10.1121/1.4979336 28464619

[B38] Li H. N. Chau C. K. Tang S. K. (2010). Can surrounding greenery reduce noise annoyance at home? *Sci. Total Environ.* 408 4376–4384. 10.1016/j.scitotenv.2010.06.025 20638105

[B39] Macdonald J. S. Lavie N. (2011). Visual perceptual load induces inattentional deafness. *Attent. Percept. Psychophys.* 73 1780–1789. 10.3758/s13414-011-0144-4 21611856 PMC3152714

[B40] Mack A. Rock I. (1998). *Inattentional Blindness.* Cambridge: MIT Press.

[B41] Maffei L. Masullo M. Aletta F. Di Gabriele M. (2013). The influence of visual characteristics of barriers on railway noise perception. *Sci. Total Environ.* 445 41–47. 10.1016/j.scitotenv.2012.12.025 23314121

[B42] Miedema H. M. Vos H. (2003). Noise sensitivity and reactions to noise and other environmental conditions. *J. Acoust. Soc. Am.* 113 1492–1504. 10.1121/1.1547437 12656384

[B43] Miller Z. D. Hallo J. C. Sharp J. L. Powell R. B. Lanham J. D. (2014). Birding by ear: a study of recreational specialization and soundscape preference. *Hum. Dimens. Wildl.* 19 498–511. 10.1080/10871209.2014.921845

[B44] Miller Z. D. Taff B. D. Newman P. (2018). Visitor experience of wilderness soundscapes in Denali national Park and Preserve. *Int. J. Wilderness* 2.

[B45] Musacchia G. Sams M. Skoe E. Kraus N. (2007). Musicians have enhanced subcortical auditory and audiovisual processing of speech and music. *Proc. Natl. Acad. Sci. U.S.A.* 104 15894–15898. 10.1073/pnas.0701498104 17898180 PMC2000431

[B46] Neisser U. Becklen R. (1975). Selective looking: attending to visually specified events. *Cogn. Psychol.* 7 480–494. 10.1016/0010-0285(75)90019-5

[B47] O’Shea D. M. Fieo R. A. (2015). Individual differences in fluid intelligence predicts inattentional blindness in a sample of older adults: a preliminary study. *Psychol. Res.* 79 570–578. 10.1007/s00426-014-0594-0 25001000

[B48] Pammer K. Korrel H. Bell J. (2014). Visual distraction increases the detection of an unexpected object in inattentional blindness. *Vis. Cogn.* 22 1173–1183. 10.1080/13506285.2014.987859

[B49] Payne S. R. (2013). The production of a perceived restorativeness soundscape scale. *Appl. Acoust.* 74 255–263. 10.1016/j.apacoust.2011.11.005

[B50] Pilcher E. J. Newman P. Manning R. E. (2009). Understanding and managing experiential aspects of soundscapes at Muir Woods National Monument. *Environ. Manag.* 43 425–435. 10.1007/s00267-008-9224-1 19020928

[B51] Sandhu R. Dyson B. J. (2016). Cross-modal perceptual load: the impact of modality and individual differences. *Exp. Brain Res.* 234 1279–1291. 10.1007/s00221-015-4517-0 26670905

[B52] Scialfa C. T. Esau S. P. Joffe K. M. (1998). Age, target-distractor similarity, and visual search. *Exp. Aging Res.* 24 337–358. 10.1080/036107398244184 9783154

[B53] Shepherd D. Heinonen-Guzejev M. Hautus M. J. Heikkilä K. (2015). Elucidating the relationship between noise sensitivity and personality. *Noise Health* 17 165–171. 10.4103/1463-1741.155850 25913556 PMC4918655

[B54] Simons D. J. Chabris C. F. (1999). Gorillas in our midst: sustained inattentional blindness for dynamic events. *Perception* 28 1059–1074. 10.1068/p281059 10694957

[B55] Sommers M. S. Tye-Murray N. Spehar B. (2005). Auditory-visual speech perception and auditory-visual enhancement in normal-hearing younger and older adults. *Ear Hear.* 26 263–275. 10.1097/00003446-200506000-00003 15937408

[B56] Sörqvist P. (2010). Effects of aircraft noise and speech on prose memory: what role for working memory capacity? *J. Environ. Psychol.* 30 112–118. 10.1016/j.jenvp.2009.11.004

[B57] Sörqvist P. Rönnberg J. (2014). Individual differences in distractibility: an update and a model. *Psych J.* 3 42–57. 10.1002/pchj.47 25632345 PMC4285120

[B58] Stansfeld S. A. (1992). Noise, noise sensitivity and psychiatric disorder: epidemiological and psychophysiological studies. *Psychol. Med. Monogr. Suppl.* 22 1–44. 10.1017/S0264180100001119 1343357

[B59] Sun K. De Coensel B. Echevarria Sanchez G. M. Van Renterghem T. Botteldooren D. (2018). Effect of interaction between attention focusing capability and visual factors on road traffic noise annoyance. *Appl. Acoust.* 134 16–24. 10.1016/j.apacoust.2018.01.001

[B60] van den Brink R. L. Cohen M. X. van der Burg E. Talsma D. Vissers M. E. Slagter H. A. (2013). Subcortical, modality-specific pathways contribute to multisensory processing in humans. *Cereb. Cortex* 24 2169–2177. 10.1093/cercor/bht069 23529004

[B61] Van Renterghem T. Botteldooren D. (2016). View on outdoor vegetation reduces noise annoyance for dwellers near busy roads. *Landsc. Urban Plan.* 148 203–215. 10.1016/j.landurbplan.2015.12.018

[B62] Wada Y. Kitagawa N. Noguchi K. (2003). Audio–visual integration in temporal perception. *Int. J. Psychophysiol.* 50 117–124. 10.1016/S0167-8760(03)00128-4 14511840

[B63] Weinstein N. D. (1978). Individual differences in reactions to noise: a longitudinal study in a college dormitory. *J. Appl. Psychol.* 63 458–466. 10.1037/0021-9010.63.4.458 701213

[B64] Weinzimmer D. Newman P. Taff D. Benfield J. Lynch E. Bell P. (2014). Human responses to simulated motorized noise in national parks. *Leis. Sci.* 36 251–267. 10.1080/01490400.2014.888022

[B65] World Medical Association (2001). World medical association declaration of Helsinki: ethical principles for medical research involving human subjects. *Bull. World Health Organ.* 79 373–374. 11357217 PMC2566407

[B66] Zhang B. Shi L. Di G. (2003). The influence of the visibility of the source on the subjective annoyance due to its noise. *Appl. Acoust.* 64 1205–1215. 10.1016/S0003-682X(03)00074-4

